# Genome-wide association and genomic prediction of resistance to maize lethal necrosis disease in tropical maize germplasm

**DOI:** 10.1007/s00122-015-2559-0

**Published:** 2015-07-08

**Authors:** Manje Gowda, Biswanath Das, Dan Makumbi, Raman Babu, Kassa Semagn, George Mahuku, Michael S. Olsen, Jumbo M. Bright, Yoseph Beyene, Boddupalli M. Prasanna

**Affiliations:** International Maize and Wheat Improvement Center (CIMMYT), P. O. Box 1041, Village Market, Nairobi, 00621 Kenya; International Maize and Wheat Improvement Center (CIMMYT), Hyderabad, India

## Abstract

*****Key message***:**

**Genome-wide association analysis in tropical and subtropical maize germplasm revealedthatMLND resistance is influenced by multiple genomic regions with small to medium effects.**

**Abstract:**

The maize lethal necrosis disease (MLND) caused by synergistic interaction of *Maize chlorotic mottle virus* and *Sugarcane mosaic virus*, and has emerged as a serious threat to maize production in eastern Africa since 2011. Our objective was to gain insights into the genetic architecture underlying the resistance to MLND by genome-wide association study (GWAS) and genomic selection. We used two association mapping (AM) panels comprising a total of 615 diverse tropical/subtropical maize inbred lines. All the lines were evaluated against MLND under artificial inoculation. Both the panels were genotyped using genotyping-by-sequencing. Phenotypic variation for MLND resistance was significant and heritability was moderately high in both the panels. Few promising lines with high resistance to MLND were identified to be used as potential donors. GWAS revealed 24 SNPs that were significantly associated (*P* < 3 × 10^−5^) with MLND resistance. These SNPs are located within or adjacent to 20 putative candidate genes that are associated with plant disease resistance. Ridge regression best linear unbiased prediction with five-fold cross-validation revealed higher prediction accuracy for IMAS-AM panel (0.56) over DTMA-AM (0.36) panel. The prediction accuracy for both within and across panels is promising; inclusion of MLND resistance associated SNPs into the prediction model further improved the accuracy. Overall, the study revealed that resistance to MLND is controlled by multiple loci with small to medium effects and the SNPs identified by GWAS can be used as potential candidates in MLND resistance breeding program.

**Electronic supplementary material:**

The online version of this article (doi:10.1007/s00122-015-2559-0) contains supplementary material, which is available to authorized users.

## Introduction

Maize lethal necrosis disease (MLND) has emerged as a devastating disease in eastern Africa since 2011 (Wangai et al. 2012). MLND in eastern Africa was found to result from synergistic interaction between *Maize chlorotic mottle virus* (MCMV) and S*ugarcane mosaic virus* (SCMV). Although each of these viruses individually can cause disease, the synergistic interactions are more pronounced. SCMV was reported in Kenya many years ago (Louie 1980). MCMV was first identified in Peru in 1973 (Castillo and Hebert 1974) and has been subsequently reported in the USA, parts of Latin America, and China (Niblett and Clafin 1978; Uyemoto et al. 1980; Xie et al. 2011). Wangai et al. (2012) first reported the MLND and MCMV in Kenya since the MLND has been reported in Uganda, Tanzania, Democratic Republic of the Congo, South Sudan and Ethiopia, seriously threatening maize production and the livelihoods of smallholder farmers in eastern Africa (Adams et al. 2013, 2014).

Maize plants are susceptible to MLND at all growth stages, from seedling to maturity. The diagnostic symptoms of MLND include chlorotic mottling of leaves, necrosis development from the leaf margin to the midrib, and dead heart; later-stage infection could lead to sterile pollen, small cobs with poor seed set, or death of the plants. Possible factors that contributed to the devastating effect of MLND in eastern Africa include new and perhaps highly virulent strains of MCMV and SCMV, conducive environment for survival and spread of insect-vectors of the two viruses (Cabanas et al. 2013), conducive environment for proliferation of the insect vectors of the two viruses, and continuous maize cropping in certain regions leading to build-up of virus inoculum. Studies undertaken jointly by International Maize and Wheat Improvement Center (CIMMYT) and Kenya Agriculture and Livestock Research Organization (KALRO) since 2012 revealed the vulnerability of a large array (nearly 90 percent) of pre-commercial and commercial maize germplasm to the MLND, especially under artificial inoculation. The maize seed industry in eastern Africa is under significant pressure to quickly replace the highly vulnerable commercial hybrids. Therefore, accelerated development and deployment of improved maize varieties with resistance to MLND is now a top priority in eastern Africa. This in turn requires intensive screening of germplasm for identifying sources of resistance, understanding the genetic architecture of MLND resistance, and utilizing molecular markers in breeding programs for fast-tracking development of improved varieties with MLND resistance and other relevant traits for the African smallholder farmers.

Genome-wide association study (GWAS) enables analysis of genetic architecture of complex traits (Yan et al. 2011). Compared to traditional linkage mapping, GWAS offers higher resolution and greater ability for identifying favorable genetic loci responsible for the trait of interest, while saving cost and time (Yu and Buckler 2006). Linkage disequilibrium (LD) decay is rapid in maize due to its high diverse nature. Therefore, large numbers of polymorphic SNPs are required to ensure complete coverage of the genome. Genotyping-by-sequencing (GBS) generates millions of SNPs with affordable cost. To date, GWAS has been successfully applied to identify quantitative trait loci (QTL) or genomic regions conferring resistance to some important diseases of maize, such as Fusarium ear rot (Zila et al. 2013), gray leaf spot (Shi et al. 2014), head smut (Weng et al. 2012), Northern corn leaf blight (Poland et al. 2011), Southern corn leaf blight (Kump et al. 2011), and SCMV (Tao et al. 2013). However, GWAS has not yet been undertaken or reported for identifying genomic regions influencing resistance to MLND.

Genomic selection or genome-wide selection (GS) is another promising breeding tool to improve the efficiency and speed of the breeding process (Zhao et al. 2012; Beyene et al. 2015). GS involves use of a ‘training population’ of individuals that have been phenotyped and genotyped, for developing the prediction model. In the next step, this model is used to predict genomic estimated breeding values (GEBVs) of the individuals from the ‘estimation set’ which are not phenotyped but genotyped with high-density markers (Meuwissen et al. 2001). Initial GS studies applied to maize agronomic traits like plant height and dry matter yield showed promising results with high prediction accuracies (Riedelsheimer et al. 2012; Zhao et al. 2012). The prediction accuracies on complex diseases like Northern corn leaf blight resistance (Technow et al. 2013) and Fusarium ear rot (Zila 2014) in maize clearly indicated the potential of GS for improving quantitative disease resistance. This motivated us to implement GS on a complex trait like MLND.

In this study, two association mapping (AM) panels, namely IMAS (Improved Maize for African Soils) and DTMA (Drought Tolerant Maize for Africa), were used for understanding the genetic architecture of MLND resistance. The objectives of the study were (1) to evaluate the diverse array of tropical and subtropical maize lines for their responses to MLND under artificial inoculation; (2) to identify genomic regions, SNPs, and putative candidate genes associated with MLND resistance; and (3) to assess the potential of GS for MLND resistance in maize.

## Materials and methods

### Plant materials and field trials

Two AM panels constituted under two major projects in Sub-Saharan Africa, namely DTMA (Drought Tolerant Maize for Africa) and IMAS (Improved Maize for African Soils), led by the Global Maize Program of the CIMMYT were used in this study. The IMAS-AM and DTMA-AM panels comprised 380 and 235 lines, respectively, representing broadly the tropical/subtropical maize genetic diversity, including germplasm derived from breeding programs targeting tolerance to drought, soil acidity, and low N, resistance to insects and pathogens (Wen et al. 2011).

### Collection and maintenance of virus isolates

Stock isolates of MCMV and SCMV were collected from MLN hotspot areas in Kenya. Once confirmed on the presence of SCMV or MCMV by Enzyme-linked immunosorbent assay (ELISA), both viruses were propagated on a susceptible hybrid, H614, in separate greenhouses. Infected leaf samples collected from the field were cut into small pieces and ground in a mortar and pestle in grinding buffer (10 mM potassium-phosphate, pH 7.0). The resulting sap extract was centrifuged for 2 min at 12,000 rpm. Carborundum was added to decanted sap extract at the rate of 0.02 g/ml. The susceptible hybrid H614 at two leaves stage was inoculated by rubbing sap extract onto the leaves. Two separate, sealed greenhouses were maintained for SCMV and MCMV inoculum production. Three weeks before inoculation, ELISA was undertaken on random samples of leaves from the SCMV and MCMV greenhouses to confirm the inoculum purity.

### Artificial field inoculation and phenotyping

In order to keep uniform MLND pressure across field trials, the optimized combination of SCMV and MCMV viruses (ratio of 4:1) were mixed and inoculated twice at 5th and 6th week after planting. Plants were inoculated using a motorized, backpack mist blower (Solo 423 MistBlower, 12 L capacity). An open nozzle (2-inche diameter) was used to deliver inoculum spray at a pressure of 10 kg/cm^2^. The presence of both viruses in the field trials was confirmed by ELISA once disease symptoms were apparent (approximately 2-week post-inoculation).

All inbred lines were evaluated in one-row 3 m plots with two replications in alpha lattice design in three seasons during 2012–2014 at Narok [latitude 01°05′S, longitude 35°52′E, 1827 m above sea level (asl)] and Naivasha (latitude 0°43′S, longitude 36°26′E, 1896 m asl) in Kenya. All standard agronomic management practices were followed. Disease severity was scored for MLN at three-week post-inoculation. Inbreds were rated visually on a 1–5 disease severity scale, where 1 = no visible MLN symptoms, 2 = fine chlorotic streaks mostly on older leaves, 3 = chlorotic mottling throughout the plant, 4 = excessive chlorotic mottling on lower leaves and necrosis of newly emerging leaves (dead hear), and 5 = complete plant necrosis.

### Phenotypic data analyses

For data based on ordinal scales, it is important to evaluate whether the data meets the assumptions of the applied statistical model (independent, normally distributed and constant variance; Rawlings et al. 1998). In this study for both the panels, we plotted the residuals against predicted values which revealed that the variance was constant. The histogram plot of the residuals was slightly deviated from normal distribution in DTMA panel compared to IMAS panel (data not shown). Therefore, we used the original data for the analyses without any transformation. Analyses of variance within and across environments was determined by the restricted maximum likelihood method using SAS 9.2 (SAS Institute 2010). Variance components were estimated by following linear mixed model: *Y*_*ijko*_ = *µ* + *g*_*i*_ + *l*_*j*_ + *r*_*kj*_ + *b*_*ojk*_ + *e*_*ijko*_, where *Y*_*ijko*_ was the phenotypic performance of the *i*th genotype at the *j*th environment in the *k*th replication of the *o*th incomplete block, *µ* was an intercept term, *g*_*i*_ was the genetic effect of the *i*th genotype, *l*_*j*_ was the effect of the *j*th environment, *r*_*kj*_ was the effect of the *k*th replication at the *j*th environment, *b*_*ojk*_ was the effect of the *o*th incomplete block in the *k*th replication at the *j*th environment, and *e*_*ijko*_ was the residual. Environments and replications were treated as fixed effects and the other effects as random. Heritability on an entry-mean basis was estimated from the variance components as the ratio of genotypic to phenotypic variance. In addition, best linear unbiased estimates (BLUEs) were estimated across environments assuming fixed genotype effects. For association analyses, best linear unbiased prediction (BLUP) of each line was calculated for across environments.

### Molecular data analyses

DNA of all inbred lines was extracted from greenhouse-grown seedlings at 3–4 leaves stage. DNA was used for genotyping using GBS platform (Elshire et al. 2011) at Cornell University, Ithaca, USA, as per the procedure described in earlier studies (Elshire et al. 2011; Glaubitz et al. 2014). For quality screening in both the AM panels, SNPs which were either monomorphic, had missing value of >5 %, heterozygosity of >5 %, or had a minor allele frequency of <0.02 were discarded from the analysis. After these quality checks, 259,000 and 264,000 high-quality SNPs were retained for GWAS in the IMAS and DTMA-AM panels, respectively.

### Genome-wide association study (GWAS)

BLUP of each line was used as phenotypes in AM scans. MLND severity data were corrected for population structure using general linear model (GLM), as well as population structure and kinship (Q + K) using mixed linear model (MLM) algorithm (Flint-Garcia et al. 2005; Yu and Buckler, 2006). GWAS and principal component (PC) analysis was performed using TASSEL ver 4.0 (Bradbury et al. 2007). The first three PCs were used to correct the population structure. The threshold *P* value (*P* < 3 × 10^−5^) was determined by considering the pattern of the Q–Q plot of the model and the point at which the observed *F* test statistics deviated from the expected *F* test statistics (Gao et al. 2010; Sukumaran et al. 2012, 2015). The total proportion of phenotypic variance explained by the detected QTL was calculated by fitting all significant SNPs simultaneously in a linear model to obtain $R_{\rm {adj}}^2$. The proportion of the genotypic variance explained by all QTL was calculated as the ratio of $p_G = R_{\rm {adj}}^2/h^2$. The 60 bp source sequences of the significantly associated SNPs were used to perform BLAST searches against the ‘B73’ RefGen_v2 (http://blast.maizegdb.org/home.php?a=BLAST_UI). Within the local LD block including associated SNPs, the filtered genes in MaizeGDB (http://www.maizegdb.org) containing directly or adjacent to each associated SNP were considered as possible candidate genes for MLND resistance.

### Genomic selection

Ridge regression best linear unbiased prediction (RR-BLUP; Whittaker et al. 2000) was applied on the BLUEs across environments. From the GBS SNP marker data, a sub-set of 2000 SNPs distributed uniformly across genome, with no missing values, and minor allele frequency >0.05 were used for genomic prediction in both the AM panels. Details of the implementation of the RR-BLUP model were described by Zhao et al. (2012). Prediction accuracy of the GS approach was evaluated using the five-fold cross-validation with 1000 times repetitions. The correlation between observed and predicted phenotypes (*r*_MP_) was estimated. The accuracy of GS was calculated as *r*_GS_ = *r*_MP_/h (Dekkers 2007), where h refers to the square root of heritability. The genomic prediction was carried out in two scenarios where both the training and estimation populations were derived from (1) within AM panels, (2) across AM panels. Additionally, for both the scenarios, prediction was carried out with and without inclusion of GWAS based MLND resistance associated SNPs. In GS, optimizing the number of markers and the training population size without losing accuracy is crucial. Therefore, we checked the effect of prediction accuracy with different number of SNPs varying from 300 to 14,000, and the number of individuals from 20 to 100 % with the interval of 20 % of the total population size.

## Results

**Fig. 1:**
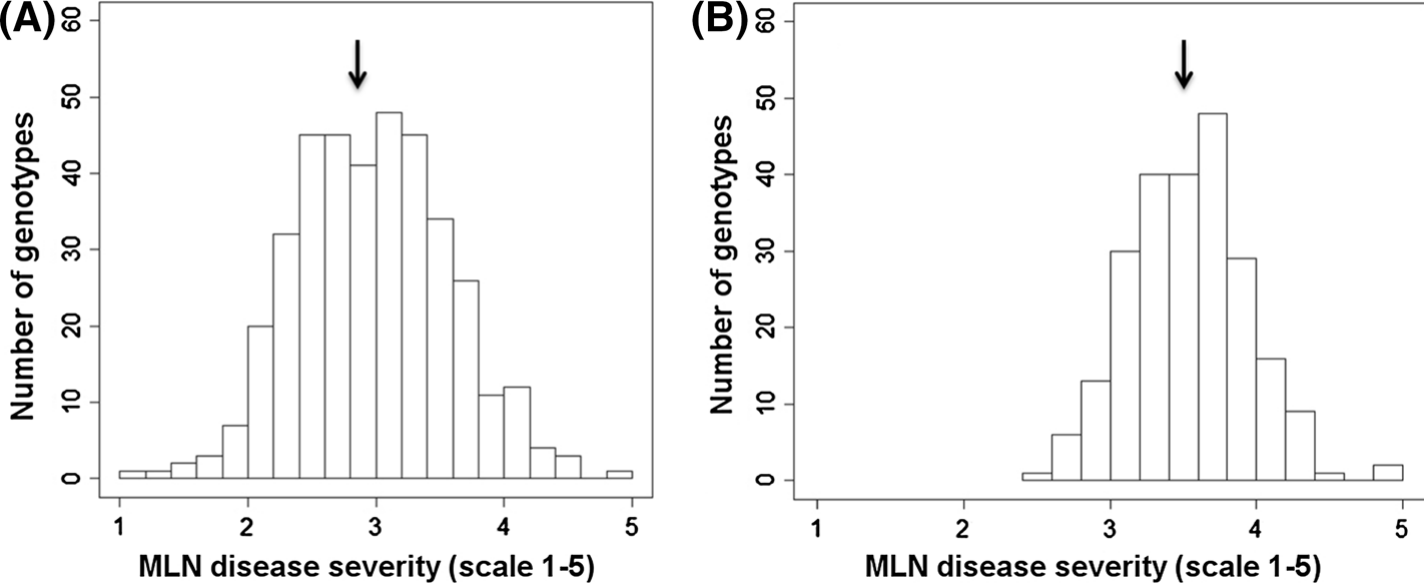
Phenotypic distribution of MLND scores on a 1–5 scale in the IMAS-AM (A) and DTMA-AM (B) panels (mean values are indicated by *arrows*)

In each environment, average MLND severity rate was higher for the DTMA-AM panel, compared to the IMAS-AM panel (Supplementary Figure S1). For both the panels, moderate yet significant correlations were observed among the genotypic values estimated in each environment (Supplementary Table S1). This ruled out the possible bias due to environment-specific disease responses in a combined analysis. Analysis across environments revealed higher average diseases severity in DTMA-AM panel (3.53) than IMAS-AM panel (2.98) in 1–5 disease scale (Table 1). The frequency of the phenotypic values in both the panels followed approximately a normal distribution with larger range of distribution for IMAS panel (Fig. 1). The ANOVA across environments revealed significant genotypic and genotype × environment interaction variances for MLND responses in both the panels (Table 1). The estimate of heritability was high with 0.73 for IMAS and 0.62 for DTMA panel, which reveals predominance of additive control of responses of maize genotypes to MLND resistance.

**Table 1:** Means, ranges, genotypic variance components (σ_G_^2^), error variances (σ_e_^2^), and broad sense heritability’s (*h* ^2^) of 380 lines of IMAS-AM panel, and 235 lines of DTMA-AM panel evaluated for MLND on a 1–5 scale in individual and across environments

Trait-MLND	Environment	Mean (range)	σ_G_^2^	σ_G × E_^2^	σ_e_^2^	*h* ^2^
IMAS-AM panel	Narok-2012	2.84 (1.00–4.99)	0.36**	–	0.27	0.73
	Narok-2013	3.20 (0.92–5.00)	0.34**	–	0.32	0.68
	Naivasha-2013	2.83 (1.14–5.00)	0.25**	–	0.60	0.40
	Across environments	2.98 (1.15–4.85)	0.25**	0.08**	0.38	0.73
DTMA-AM panel	Naivasha-2013	3.66 (2.01–5.00)	0.16**	–	0.19	0.63
	Naivasha-2014	3.29 (1.99–4.25)	0.11**	–	0.22	0.68
	Narok-2013	3.61 (2.31–5.00)	0.12**	–	0.34	0.41
	Across environments	3.53 (2.51–5.00)	0.09**	0.05**	0.23	0.62

** Significant at *P* < 0.01

Among 615 lines evaluated for MLND response, 14 lines were selected as best performing lines (Table 2). Interestingly yellow lines derived from tropical lowland breeding programs from Mexico were the best lines among the selected lines for MLND resistance. African breeding programs where white maize is predominant, and we found five lines which showed relatively better resistance for MLND.

**Table 2:** The performance of selected lines with better resistance or lower disease severity against MLND in each and across three environments

Genotype	MLND scores (scale 1–5)				Heterotic group	Seed color	Breeding program	Adaptation
	Env1	Env2	Env3	Across Env				
CLRCY039	1.18	0.92	1.14	1.17	B	Yellow	CIMMYT lowland tropics	Tropical lowlands
CPHYS138	1.02	1.49	1.42	1.32	A	Yellow	CIMMYT Physiology	Lowland/subtropical
CLRCY034	1.14	1.62	1.71	1.48	B	Yellow	CIMMYT lowland tropics	Tropical lowlands
CLWN270	1.36	1.80	1.41	1.52	AB	Yellow	CIMMYT lowland tropics	Tropical lowlands
CKL05003	1.12	1.58	2.12	1.62	B	White	CIMMYT Kenya	Africa mid-elevation/subtropical
SM-189-75	1.07	1.57	2.26	1.69	–	Orange	KALRO, Kenya	Mid-elevation
CLWQ251	1.42	2.28	1.60	1.81	B	White	CIMMYT lowland tropics	Tropical lowlands
CML494	1.33	2.30	1.92	1.83	AB	White	CIMMYT Gene bank	Lowland
SM-189-38	1.73	1.88	1.63	1.86	–	White	KALRO, Kenya	Highland
CPHYS159	1.97	1.50	2.15	1.86	A	White	CIMMYT Physiology	Lowland
CLYN261	1.87	1.49	2.19	1.87	A	Yellow	CIMMYT lowland tropics	Tropical lowlands
SM-189-78	1.86	2.75	1.14	1.88	–	Orange	KALRO, Kenya	Mid-elevation
CLYN231	1.08	2.03	2.48	1.90	A	Orange	CIMMYT lowland tropics	Tropical lowlands
SM-189-69	1.23	1.98	2.59	1.99	–	Yellow	KALRO, Kenya	Mid-elevation

**Fig. 2:**
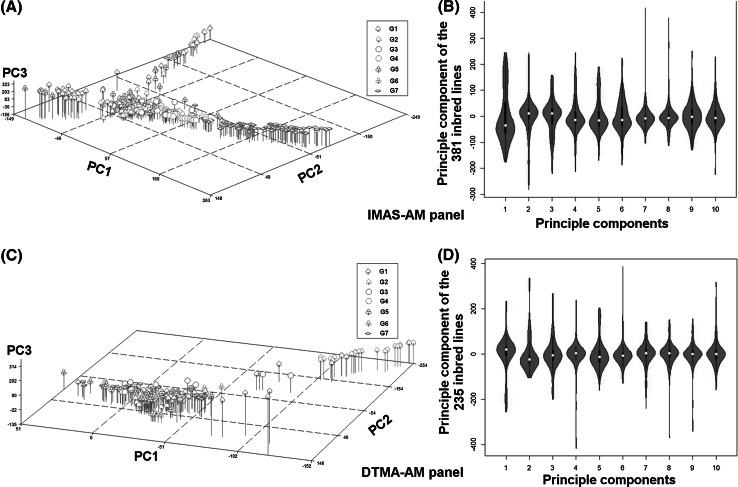
Population structure based on principal component (PC) analysis of IMAS-AM (**a**) and DTMA-AM (**c**) panels. Violin plot showing the density distribution of the first ten principal components for the genotypes from IMAS-AM (**b**) and DTMA-AM (**d**) panel. (In the IMAS-AM panel, the seven groups represent lines from the breeding program of *G1* CIMMYT gene bank, *G2* Physiology, *G3* Zimbabwe, *G4* Kenya, *G5* Lowland tropical, *G6* MAS-DT, and *G7* ARC South Africa; and in the DTMA-AM panel, the seven groups represent lines from the breeding program of *G1* Tropical, *G2* Physiology, *G3* Zimbabwe, *G4* Kenya, *G5* Subtropical, *G6* Entomology, and *G7* Columbia)

Principal component analysis revealed the presence of a clear population structure in both the panels with respect to first three PCs (Fig. 2a, c), as well as by several of the first ten PCs as revealed by their density distribution (Fig. 2b, d). In IMAS panel, lines derived from the CIMMYT physiology program and from the South African Agriculture Research Council’s (ARC’s) breeding program formed distinct clusters (Fig. 2a). In the DTMA panel too, the lines developed by CIMMYT physiology program formed a distinct group (Fig. 2c).

**Fig. 3:**
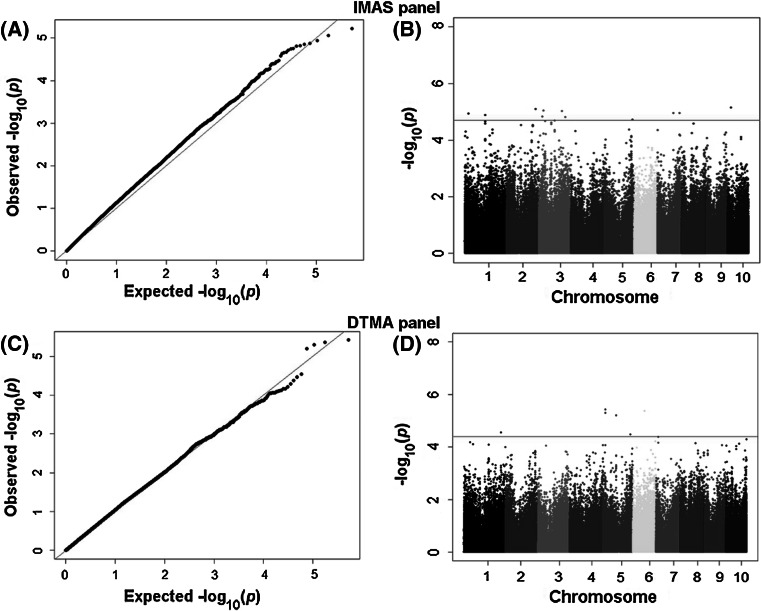
Quantile–quantile plots (**a**, **c**), and Manhattan plots of a mixed linear model for MLND resistance in the IMAS-AM and DTMA-AM panels. Plots above *red horizontal line* showed the genome-wide significance with stringent threshold of *P* = 3 × 10^−5^. The *different colors* indicate the 10 different chromosomes of maize (color figure online)

From the GBS data, we selected a set of ~260 K high-quality polymorphic SNPs for GWAS. Manhattan plots of the GWAS results for both IMAS and DTMA panels are shown in Fig. 3. In the IMAS-AM panel, we detected 18 significant marker–trait associations for MLND resistance (Table 3, *P* < 3 × 10^−5^). These significantly associated SNPs individually explained 8–10 % of the total genotypic variance, whereas together explained 30 % of the total proportion of genotypic variance for resistance to MLND. In the DTMA-AM panel, we detected six significant marker–trait associations which individually explained 14–18 % of the total genotypic variance and together explained 37 % of the total proportion of genotypic variance for MLND resistance (Table 4). Comparison of the significant SNPs in the two AM panels revealed that there were no common marker–trait associations across panels; however, there was some similarity on number of SNPs falling into same chromosome bins. We used B73 maize genome reference sequence to identify putative candidate genes based on the SNPs significantly associated with MLND resistance (Tables 3, 4). From both the AM panels, a set of putative candidate genes were identified; based on their functions, these can be grouped as either R genes or plant defense responsive genes.

**Table 3:** Details of the MLND resistance associated SNP markers identified in the IMAS association mapping panel

SNP	Chr	Position	MLM-*P* values	*P*_G_ (%)	MAF	Allele	Allele effect	Putative candidate genes	Predicted function of candidate gene
S10_23785810	10	23,785,810	7.08E−06	8.95	0.03	A/G	0.60	GRMZM2G451231	Unknown
S2_211771737	2	211,771,737	8.19E−06	8.99	0.03	T/G	−1.40	GRMZM2G056612	Serine/threonine protein kinase
S3_34036135	3	34,036,135	8.96E−06	9.18	0.17	T/G	−0.01	GRMZM2G094523	Plant-type cell wall organization
S3_165911594	3	165,911,594	9.38E−06	8.57	0.09	A/G	0.58	GRMZM2G177244	REM Transcription Factor
S7_115310293	7	115,310,293	1.12E−05	8.75	0.20	C/T	0.44	GRMZM2G125653	WRKY DNA-binding protein
S7_158464599	7	158,464,599	1.14E−05	8.59	0.27	C/A	0.30	GRMZM2G006942	Virus induced gene silencing
S1_24941000	1	24,941,000	1.17E−05	8.61	0.28	C/T	0.31	GRMZM2G032423	Putative uncharacterized protein
S1_148453861	1	148,453,861	1.33E−05	8.64	0.04	C/T	0.26	GRMZM2G135045	Aminopeptidase activity
S3_22944526	3	22,944,526	1.46E−05	8.95	0.41	T/C	0.38	GRMZM2G471517	Antifreeze
S3_189356738	3	189,356,738	1.52E−05	8.41	0.30	C/A	0.38	GRMZM2G008109	Serine-type endopeptidase activity
S5_205339659	5	205,339,659	1.93E−05	8.25	0.02	T/C	0.83	GRMZM2G181505	Dihydroorotate dehydrogenase
S3_114355785	3	114,355,785	2.05E−05	7.95	0.21	T/C	0.33	GRMZM2G405385	Homoiothermy/antifreeze
S3_44062810	3	44,062,810	2.12E−05	8.04	0.36	C/T	0.38	GRMZM2G404316	Antifreeze
S3_90976758	3	90,976,758	2.38E−05	8.52	0.42	C/G	0.28	GRMZM2G077415	Malate dehydrogenase activity
S1_148456035	1	148,456,035	2.59E−05	8.17	0.04	A/T	0.25	GRMZM2G135045	Manganese ion binding/aminopeptidase activity
S3_90976749	3	90,976,749	2.73E−05	8.41	0.42	T/C	0.28	GRMZM2G077415	Malate dehydrogenase activity
S2_193503877	2	193,503,877	2.94E−05	8.32	0.36	C/G	−0.29	GRMZM2G150541	Cellular metabolic process/steroid biosynthetic process
S2_105760109	2	105,760,109	2.98E−05	8.24	0.15	A/G	0.13	GRMZM2G137984	Protein binding/retrograde transport endosome to Golgi
Total *P*_g_ (%)					30.14				

*MLM* mixed linear model, *MAF* minor allele frequency, *P*_*g*_ proportion of genotypic variance

^a^The exact physical position of the SNP can be inferred from marker’s name, for example, S2_211771737: chromosome 2; 211,771,737 bp

**Table 4:** Details of the MLND resistance associated SNP markers identified in the DTMA association mapping panel

SNP	Chr	Position (Mb^a^)	MLM-P values	*P*_G_ (%)	MAF	Allele	Allele effect	Putative candidate genes	Predicted function of candidate gene
S5_16839191	5	16.8	3.83E−06	18.44	0.10	C/T	−1.00	GRMZM2G018943	Translation initiation factor eIF-2B delta subunit
S6_84786872	6	84.7	4.57E−06	18.42	0.06	C/A	−0.75	GRMZM2G139073	MADS-box transcription factor
S5_16837972	5	16.8	6.09E−06	17.91	0.11	G/A	−0.99	GRMZM2G077828	Unknown
S5_95192724	5	95.1	6.12E−06	18.81	0.06	G/A	0.06	GRMZM2G109805	Hypersensitivity
S1_269037989	1	269.0	2.80E−05	16.16	0.06	C/A	−1.53	GRMZM2G047055	Actin cross link
S5_199371477	5	199.3	3.50E−05	14.91	0.03	G/T	0.88	GRMZM2G376067	MAIZE Putative uncharacterized protein
Total				37.20					

*MLM* mixed linear model, *MAF* minor allele frequency, *P*_*g*_ proportion of genotypic variance

^a^The exact physical position of the SNP can be inferred from marker’s name, for example, S2_211771737: chromosome 2; 211,771,737 bp

**Fig. 4:**
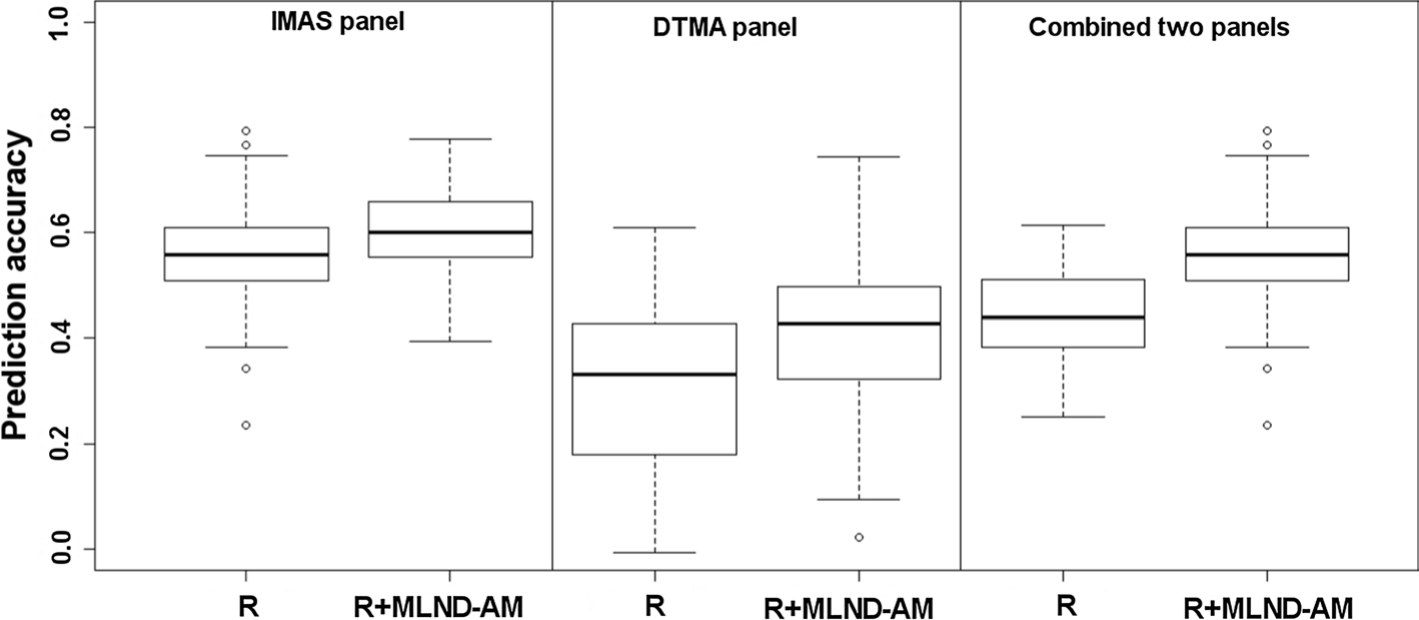
Distribution of the accuracy of genomic predictions for scenario 1 (prediction based on random markers) and scenario 2 (prediction based on random and significant MLND-associated markers) within and across IMAS-AM and DTMA-AM panels, as revealed by five-fold cross-validation for MLND resistance

**Fig. 5:**
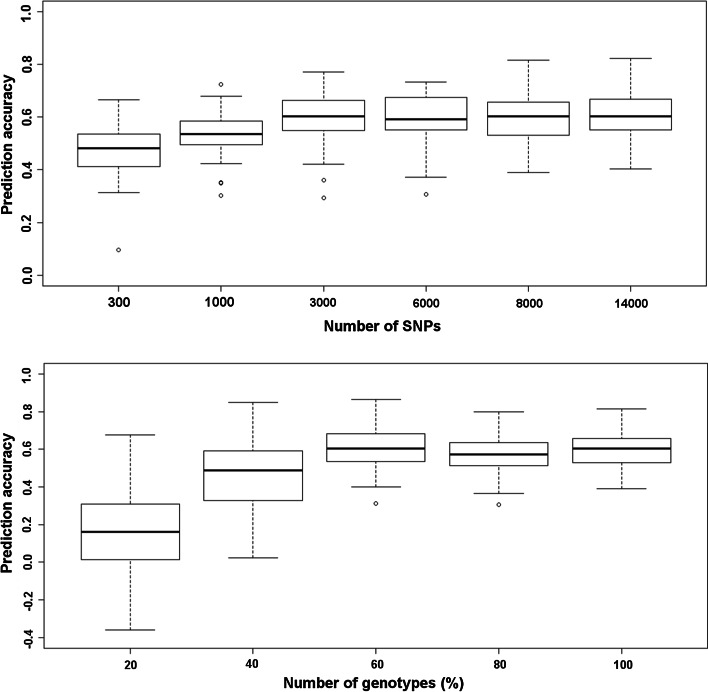
Effect of the number of markers, and the number of individuals on the accuracy of genomic prediction for MLND resistance in the IMAS association mapping panel

The accuracy of genomic predictions within the panel was higher for the IMAS-AM over the DTMA-AM panel (Fig. 4). The prediction accuracy was improved in both the panels by inclusion of the MLND resistance associated SNPs. The prediction accuracy across AM panels was 0.41 which increased to 0.56 with the inclusion of MLND resistance associated SNPs into the prediction model. The prediction accuracy was severely affected by population size, whereas the effect was relatively low with decrease in the number of markers (Fig. 5).

## Discussion

Maize lethal necrosis disease is not only due to individual effect of either SCMV or MCMV, but it also includes their interaction effects which together lead to substantial yield loss and threatening the food security currently in eastern Africa (Ali and Yan 2012). The genetics of SCMV and other potyviruses has been extensively studied in maize with diverse germplasm (as reviewed by Redinbaugh and Pratt 2009). The genetics and inheritance of MLND resistance is not known and is expected to be very complex due to combination of two viruses. GWAS and GS are the best tools used to study such complex traits (Riedelsheimer et al. 2012).

In GWAS, the power of QTL detection largely depends not only on the sample size but also on the trait architecture and heritability (Yu et al. 2008); therefore, precise phenotypic evaluation for the trait of interest is critical. To obtain reliable phenotypic data, we have used a broad array of tropical and subtropical maize germplasm and evaluated the same for MLND severity under optimized artificial inoculation procedure for three environments in Kenya. Heritability was moderately high in both AM panels. The significant genotypic variation observed in both the panels also reflected the high quality of the phenotypic data, thereby enabling identification of genomic regions with substantive power.

### Population structure and linkage disequilibrium

The lines used in this study represents various breeding programs from Kenya, Zimbabwe, South Africa, Nigeria, Malawi and Columbia, as well as from CIMMYT gene bank and some specific programs such as CIMMYT Physiology program, Latin American tropical lowland breeding program, and the mid-elevation Africa adapted breeding program (Fig. 2). As a result, confounding structure exists in these panels and false-positive associations would be expected if the data is not corrected for population structure (Yan et al. 2009). The use of first three PCs along with relative kinship matrix in the Q  +  K model enabled us to correct for spurious associations which is also evident in quantile plots (Fig. 3a, c).

The mapping resolution and the required marker density for GWAS is largely depends on the extent of LD in the population (Yu and Buckler 2006; Myles et al. 2009). The extent of LD for the panels used in this study was examined in detail in earlier study (Vinayan et al. 2013). The average *r*^2^ values between neighboring markers were 0.29 and 0.24 for IMAS-AM and DTMA-AM panel, respectively. This moderate LD estimate in both the panels suggests the diverse nature of the tropical/subtropical maize germplasm used in this study, which on the other hand leads to high mapping resolution. The observed *r*^2^ between adjacent markers was comparable to (*r*^2^ = 0.28) the earlier studies (Van Inghelandt et al. 2011; Massman et al. 2013). Although it is estimated that at least one million SNPs are required to efficiently detect all minor QTL (Gore et al. 2009), the observed average LD estimates in our study indicates that at least medium to large effect QTLs should be detected.

### Genome-wide association study for main effect QTL

Twenty-four SNPs significantly associated with MLND resistance are localized to eight out of ten different chromosomes (Tables 3, 4). In IMAS-AM panel, the total genotypic variance explained by each significantly associated SNPs was <10 %, consequently each of the QTL defined by these SNPs can be regarded as relatively minor QTL. On the contrary in DTMA-AM panel, we observed all six detected QTL explained >10 % of the total genotypic variance.

In IMAS-AM panel, eight SNPs detected on chromosome 3 are localized to the linkage map bins 3.04 and 3.05 which reportedly had resistance genes to multiple potyviruses, including SCMV, MDMV (Maize dwarf mosaic virus), MCDV (Maize chlorotic dwarf virus), MSV (Maize mosaic virus), and WSMV (Wheat streak mosaic virus; Lübberstedt et al. 2006; Jones et al. 2011; Zambrano et al. 2014). In addition, these two genomic regions are also found to confer resistance to other fungal diseases like Southern corn leaf blight, Northern corn leaf blight and gray leaf spot (Belcher 2009). Comparison of the GWAS detected SNPs position with previous QTL studies revealed that the SNP S2_211771737 was overlapped with the MMV resistance QTL(Zambrano et al. 2014). Overall coincidence of MLND resistance associated SNPs with several other virus resistance loci supports the clustering nature of QTL for multiple virus resistance. In conclusion, the identified SNPs can be used as diagnostic markers, and targeted selection of these SNPs alleles are useful in improvement of MLND resistance levels in elite breeding lines.

### Putative candidate genes

Putative candidate genes identified on chromosomes 2 and 3 were primarily involved in cell-to-cell transport of micro and macromolecules (Tables 3, 4). Plant viruses need to be able to move between mesophyll cells and also in and out of phloem tissue for systematic infection. It is assumed that plants resist the virus infection by controlling the virus movement inside the host and this mechanism is clearly demonstrated by RTM system in *Arabidopsis* (Chrisholm et al. 2000). Similarly, there is high probability that the putative candidate genes identified in this study might be involved in MLND resistance/vulnerability by controlling the movement of one or both the viruses in the plants; however, it needs to be confirmed by independent validation studies.

The plant defence mechanism against viruses is mediated by resistance (R) genes and is well characterized in several crop plants (Spassova et al. 2001; Stange et al. 2004; Vidal et al. 2002). In maize, two NBS-LRR genes are mapped into bin 3.05 of chromosome 3 (Xiao et al. 2007). On the other hand, the R genes often express complete resistance in the form of hypersensitive response by which the infected cells are killed by programmed cell death. In line with this observation, we identified one candidate gene GRMZM2G109805 on chromosome 5 which directly involved in hypersensitive reaction. Clear hypersensitive reaction and leaf death symptoms were also observed in MLND infected plants which suggest the possible role of these genes in plants resistance against viruses.

For viruses, host factors are important to complete their life cycle. Mutations in these host factors forms a recessive inherited virus resistance genes. We found one candidate gene GRMZM2G018943 functions as a translation initiation factor eIF-2B is also due to similar type of mutations. Previously, few recessive inherited virus resistance genes were also reported for potyvirus and other viruses (Ingvardsen et al. 2010). These recessive genes contribute for certain level of resistance to MLND by associating with other minor QTL of SCMV or MCMV. However, it should be point out that these candidate genes should be further validated before integrating them in a breeding program.

Two candidate genes with putative protein serine/threonine kinase activity have a role in signaling interactions during the perception of pathogens and consequent activation of defence responses (Romeis et al. 2000; Zhou et al. 1995). Three identified putative candidate genes with ice-binding functions are type of antifreeze proteins which belongs to group of pathogenesis-related proteins (Griffith and Yaish 2004; Hon et al. 1995) indicating their possible role in plant defence against MLND.

### Genomic selection

The results from this study give first insights into the potential of genome-based prediction of MLND resistance in maize. The potential of GS has been assessed for simple and complex traits in maize (Crossa et al. 2010, 2013; Zhao et al. 2012). GS allows capture contribution of even small effect QTL and lead to high prediction accuracy. Using a cross-validation approach, genomic predictions explained ~56 and ~36 % of the variation in IMAS-AM and DTMA-AM panel, respectively. This is in accordance with the previous study for complex disease like Northern corn leaf blight (Technow et al. 2013). The differences in the prediction accuracy between two AM panels can be attributed to their sample size, genetic variance, and trait heritability (Table 1). On the other hand, the differences may also reflect the changes in population structure and LD estimates. Surprisingly, prediction accuracy across panel was lower than IMAS panel which might be attributed to higher magnitude of genotypic variance observed for within panel than across panel (data not shown). Inclusion of MLND-associated SNPs into training population led only slight increase in the prediction accuracy in both the panels, indicating that prediction accuracy is mainly attributable to many small effects QTL distributed across genome.

Routine implementation of GS in breeding program is affected by resource allocation especially on cost of genotyping and phenotyping. RR-BLUP is known to perform well under low marker density (Habier et al. 2007), and accordingly, we observed marginal decrease in prediction accuracy when number of markers were reduced from 14,000 to 1000 (Fig. 5). Our finding also corroborates the earlier studies in maize (Zhao et al. 2012). However, accuracy was severely affected with the decrease in the size of training population. This clearly suggests the need of optimum size of training population which approximates *n* ~ 230 for MLND in IMAS-AM panel in the current study; however, this could vary depending on the germplasm used and the trait under study.

Possible routine use of GS in breeding for resistance to MLND depends on its relative advantage over phenotypic selection. Phenotypic selection accuracy, estimated as *h*, was 0.85 and 0.79 for IMAS and DTMA-AM panels, respectively. However, in maize, up to three cycles of GS per year are possible (Lorenzana and Bernardo 2009). Therefore, compared to phenotypic selection, GS would be more efficient in terms of genetic gain per year than per cycle.

## Conclusion

In this study, we used two AM panels together comprised 615 lines to understand the genetic architecture of MLND resistance in tropical and subtropical maize germplasm. GWAS scan identified 24 SNPs associated with resistance to MLND. GS results revealed higher selection gain per year for marker-based selection compared to phenotypic based selection for MLND resistance. Further research is warranted on validating the effects of the identified candidate genes and their functional variants to confirm that these genes engender resistance to MLND in maize. We identified few lines which can serve as a potential donor in improving susceptible commercial lines into MLND resistant lines either through marker-assisted recurrent selection or GS.

### Author contribution statement

BD, DM, GM, BMP, and MG—conceived the experiment; BD, GM, MG, and DM—conducted the field evaluations and phenotyping; MG, KS, and RB—coordinated the GBS experiments; MG—carried out the GWAS analyses; MG, BD, DM, GM, MO, BMP, JMB, KS, and RB—interpreted the results and drafted the manuscript.

## Electronic supplementary material

Supplementary material 1 (DOCX 85 kb)

## Abbreviations

MLND: Maize lethal necrosis disease
GBS: Genotyping-by-sequencing
LD: Linkage disequilibrium
MLM: Mixed linear model
MAF: Minor allele frequency
MCMV: Maize chlorotic mottle virus
SCMV: Sugarcane mosaic virus
ELISA: Enzyme-linked immunosorbent assay
