# Supplementary material for: Genome-wide association and genomic prediction of resistance to maize lethal necrosis disease in tropical maize germplasm
Source: Theor Appl Genet. 2015 Jul 8;128(10):1957–68. doi: 10.1007/s00122-015-2559-0 (PMC4572053; doi:10.1007/s00122-015-2559-0)
Supplement: Supplementary file 1 — Supplementary material 1 (DOCX 85 kb) [file 122_2015_2559_MOESM1_ESM.docx]

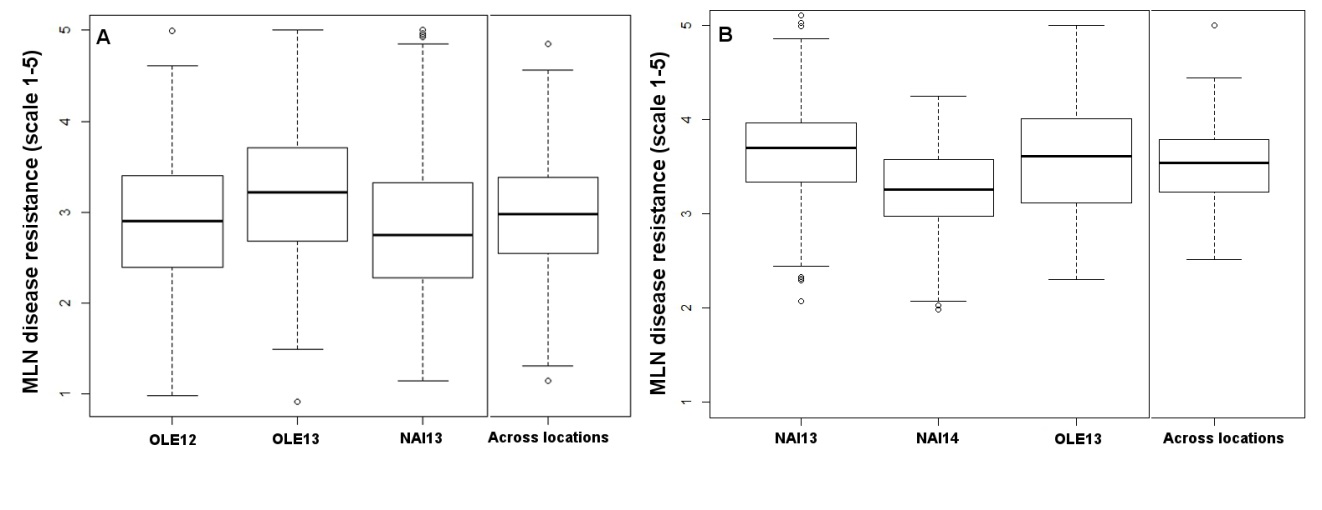


**Supplementary Figure S1**. Phenotypic distribution of MLND severity data at each and across MLND phenotyping environments in the IMAS (A) and DTMA (B) association mapping panels

**Supplementary Table S1**. Phenotypic correlations of the adjusted entry means of lines of three MLND phenotyping environments.

| IMAS panel | Narok13 | Naivasha13 |
| --- | --- | --- |
| Narok12 | 0.53** | 0.35** |
| Narok13 |  | 0.39** |
|  |  |  |
| DTMA panel | Narok13 | Naivasha13 |
| Naivasha14 | 0.34** | 0.50** |
| Narok13 |  | 0.35** |

** indicates significance at *P* < 0.01
